# Seed Oil Quality and Cultivation of *Sambucus williamsii* Hance as a New Oil Crop

**DOI:** 10.3389/fnut.2021.796175

**Published:** 2021-12-24

**Authors:** Shuyue Wang, Yongxin Yu, Mingxiao Cui, Kehai Liu, Kewu Liu

**Affiliations:** ^1^Department of Biopharmaceutics, College of Food Science and Technology, Shanghai Ocean University, Shanghai, China; ^2^National Experimental Teaching Demonstration Canter for Food Science and Engineering, Shanghai Ocean University, Shanghai, China; ^3^Mudanjiang Branch of Heilongjiang Academy of Forestry, Mudanjiang, China

**Keywords:** *Sambucus williamsii* Hance, vegetable oil, nutrition, genetic diversity, breeding

## Abstract

Natural edible oil derived from wild non-cultivated oil crops contributed to human daily nutritional diversity and disease prevention. It was important to investigate the nutritional value of these oils and the feasibility of crop cultivation. The present study focused on the assessment of seed oil quality of *Sambucus williamsii* Hance (SWH) and its molecular breeding. Wild SWH seed oil was extracted by supercritical CO_2_ technology and the composition of the oil was determined by using gas chromatography mass spectrometry (GC-MS) analysis. The oil content of SWH seeds reaches around 40%. Its seed oil was found to be rich in unsaturated fatty acids, such as 24.24% of linolenic acid and 50.56% of linoleic acid, and vitamin E (25.92 mg kg^−1^). The cytotoxicity and heavy metal analysis showed SWH seed oil was safe for consumption. In addition, the SWH strains with excellent characteristics were screened out for cultivation according to genetic diversity and morphological analysis. Amplified fragment length polymorphism (AFLP) markers were used to evaluate the genetic diversity of 28 accessions of wild SWH seeds and 5 accessions were selected to cultivate. Among them, two strains of SWH (sample 3 and 6) with high yielding (275.7 and 266.8 area yield kg^−1^) were suitable for dense planting and could be used to establish the raw material forest of SWH seed oil. The results of this study indicated the potential of development of selected SWH as novel oil crops and their wide cultivation.

## Introduction

In recent years, wild edible fruits, such as elderberries have become more and more popular. Studies have shown that they have excellent medicinal value and health functions due to their high levels of non-nutritive, nutritional, and biologically active compounds, such as flavonoids, phenolics, anthocyanins, sugars, essential oils, carotenoids, vitamins, and minerals (1–3). Bioactive compounds from wild plants have beneficial effects on human health, which showed the potential to be developed into pharmaceuticals and various nutritions. Here, we will report *Sambucus williamsii* Hance (SWH), as a widespread genus of shrubs growing in China, can be utilized to produce sufficient chemical contents to such as, lignans, terpenoids, phenolic acids, aliphatic compounds, and essential oils (4). On the other hand, public attention on oils for nutrition and pharmaceuticals is on the rise, the demands for oil-bearing fruits/crops also become important (5). Thus, there is an opportunity and challenge for us to develop more healthy edible oils to meet human needs. Oil, occupying a large proportion of daily diet of people, provides energy and calories for the human body (6). At the same time, vegetable oils are more popular than animal fats due to rich unsaturated fatty acids and vitamins, which could prevent potential diseases and have a variety of nutritional values and pharmacological activities (7–9).

One of the common characteristics of those commercially vegetable oil, such as olive, coconut, and palm oil, is a large variety of seeds which can be used for oil extraction (10, 11). In addition, SWH produces abundant seeds and the percentage of oil extracted from the seed takes up to 35–44% (12). SWH seed oil is a transparent orange-yellow liquid with strong flavor, which has a long history of consumption in folk (13). As early as more than 30 years ago, *Sambucus williamsii* (SW) seed oil had been extracted using traditional methods and consumed by Europeans and Chinese. It is also rich in fatty acids, especially linolenic acid, and vitamin E. In the previous study of our group, the oil was extracted by high pressure fluid and *in vitro* and *in vivo* biological activities were systematically studied (12). SWH seed oil not only has significant antioxidant, hypoglycemic, and lipid-lowering activities, but also has anti-cancer, anti-virus, anti-bacterial, and immune activities (14–19). Therefore, SWH oil could be expected to be a potential ideal edible vegetable oil. However, the specific nutritional value and edible safety evaluation of SWH seed oil are lacking.

*Sambucus williamsii* provides people with high-quality edible oil, but it is mainly wild at present, and its oil yield is limited, which is difficult to meet demand of people in the future. Therefore, screening the excellent species of SW and developing artificial cultivation technology will be conducive to develop it into oil crops. SWH has strong adaptability to climate—not a warm and humid environment but the drought and cold, the barren and mild saline-alkali soil not inhibiting its growth (7–9). Accordingly, SWH grows in China from north to south and east to west in various climatic zones. SWH breeding in the market is mostly based on morphological characteristics now. But this method is difficult to distinguish similar varieties and brings inconvenience to the large-scale introduction and cultivation of SWH (20, 21). If the genetic diversity of SWH germplasm resources in different habitats were distinguished and evaluated by molecular markers, the selection of SWH with better characteristics that could be artificially cultivated in various regions (22–24).

The purpose of this study was to assess the nutritional value and safety of SWH seed oil to further confirm its edible properties and to screen out wild SWH strains with excellent characteristics. It will be helpful for SWH seed oil development as a new edible oil and promote the breeding process of SWH.

## Materials and Methods

### Materials

**Figure 1:**
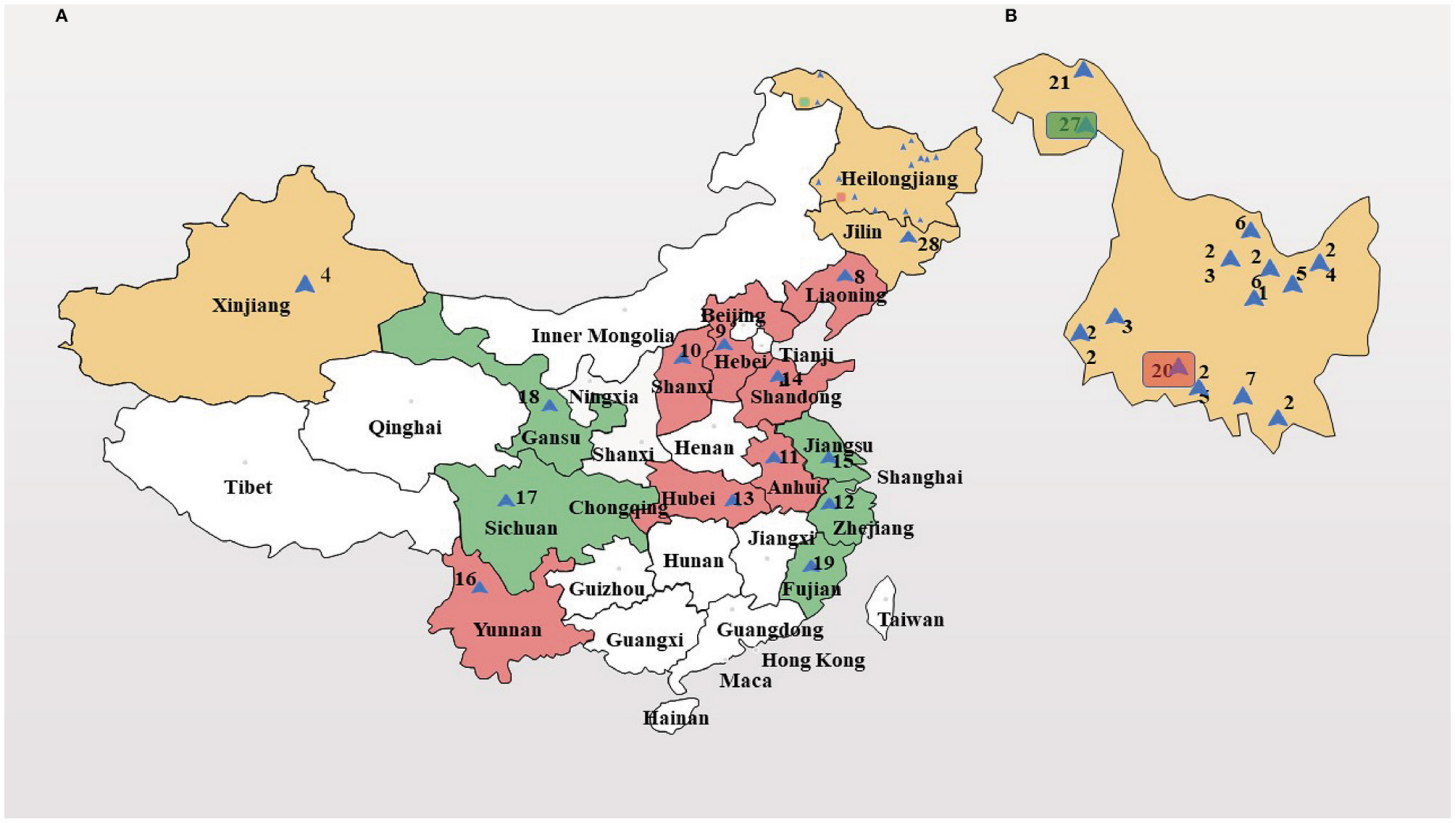
Geographic distribution of 28 *Sambucus williamsii* Hance (SWH) accessions. **(A)** map of China; **(B)** map of Heilongjiang province (enlarged). I-III: the first to third genetic group, colored by yellow, red, and green, respectively.

Wild SWH seeds were obtained from Heilongjiang Forest By-product and Speciality Institute, Heilongjiang, China. Petroleum ether, anhydrous ethanol, 95% ethanol, potassium hydroxide, methanol, n-hexane, vitamin E, diethyl ether, RPMI 1640 medium, trypsin, phosphate buffer (PBS), and dimethyl sulfoxide (DMSO) were purchased from Sinopac Chemical Reagent co., LTD (Beijing, China). Copper standard solution, lead standard solution, chromium standard solution, zinc standard solution, cadmium standard solution, mercury standard solution, and arsenic standard solution were purchased from Shanghai Mengze Biotechnology co., LTD (Shanghai, China). MTT assay kit was purchased from Shanghai Boguang biological co., LTD (Shanghai, China). Human cervical cancer cells were supplied by the Department of Pharmacy, Changhai Hospital. The DNA extraction kit was purchased from QIAGEN (Shanghai, China). MseI and EcoRI restriction enzymes, adaptors, and primers were purchased from New England BioLabs Inc. (Shanghai, China). The 28 accessions of SWH were chosen based on their sampling sites covering a diverse geographical range of China (Figure 1 and Table 1).

**Table 1:** Information of the 28 *Sambucus williamsii* Hance (SWH) tree samples and code numbers.

**Samples**	**Locations**	**Crown**	**Fruit Color**	**Latitude (E)**	**Longitude (N)**
1	Langxiang, Heilongjiang	Compact	Bright red	128°54′	47°1′
2	Ningan, Heilongjiang	Compact	Bright red	128°57′	43°51′
3	Daqing, Heilongjiang	Compact	Bright red	214°57′	46°36′
4	Xinjiang	Compact	Bright red	87°37′	43°53′
5	Daqingchuan, Heilongjiang	Moderate	Bright red	129°5′	47°1′
6	Xiaoxinganlin, Heilongjiang	Moderate	Bright red	128°54′	47°45′
7	Mudanjiang, Heilongjiang	Moderate	Bright red	129°37′	44°33′
8	Liaoning	Open	Purple red	123°33′	41°48′
9	Hebei	Open	Bright red	114°41′	38°5′
10	Shanxi	Moderate	Bright red	112°0′	38°0
11	Anhui	Open	Bright red	117°33′	32°1′
12	Zhejiang	Moderate	Purple black	120°20′	30°11′
13	Hubei	Moderate	Purple red	114°7′	30°44′
14	Shandong	Compact	Bright red	116°52′	36°21′
15	Jiangsu	Open	Purple black	119°1′	31°57′
16	Henan	Moderate	Purple red	114°1′	34°44′
17	Sichuan	Open	Purple red	103°42′	30°48′
18	Gansu	Moderate	Bright red	98°25′	39°53′
19	Fujian	Open	Purple red	119°9′	26°19′
20	Heilongjiang	Compact	Bright red	127°57′	44°37′
21	Tahe, Heilongjiang	Moderate	Bright red	124°47′	52°20′
22	Qiqihuaer, Heilongjiang	Moderate	Bright red	123°44′	47°39′
23	Wuyin, Heilongjiang	Compact	Bright red	129°24′	47°58′
24	Jixian, Heilongjiang	Compact	Bright red	130°58′	46°44′
25	YaBuLi, Heilongjiang	Compact	Bright red	128°35′	44°56′
26	Jiamusi, Heilongjiang	Moderate	Bright red	131°10′	46°43′
27	Jiagedaqi, Heilongjiang	Compact	Bright red	123°53′	50°27′
28	Jilin	Moderate	Purple red	127°22′	44°11′

### Nutritional Value Assessment of SWH Seed Oil

#### SWH Seed and Seed Oil Extraction

The SWH seeds were obtained from SWH fruits. First, the SWH fruits were slightly crushed by hands, and the crushed fruits were immersed in water. Then, the upper layer of fruits, peels, and deflated seeds were filtered and removed. The plump SWH seeds can be collected from the lower layer precipitation. Then, SWH seed oil was extracted from the laboratory by supercritical CO_2_ extraction based on previous reported method (12).

#### Fatty Acid Composition Analysis

In this study, 2 ml SWH seed oil, 2 ml mixed petroleum ether and ethanol solution (volume rate = 1:1), and 2 ml potassium hydroxide-methanol solution (0.4 mol L−1) were added to 10 ml volumetric flask. The mixture was shaken by hand for 1 min. Then, distilled water was added to the flask for constant volume after standing for 15 min. The sample for gas chromatography mass spectrometry (GC-MS) analysis was the supernatant fluid filtered by membrane with 13 mm diameter and 0.22 μm aperture. GC-MS analysis was conducted on 6890/5973N GC-MS/MS instrument (Agilent, CA, USA). GC separation was performed on a DB-5MS IU chromatographic column (30 m × 0.25 mm × 0.25 μm) and high purity helium was performed as a carrier gas at a stable flow rate with 1 ml min−1. The sample (1 μl) was injected under temperature at 230°C in split ratio of 20:1. The oven temperature program was as follows: the initial temperature was kept at 60°C for 5 min, ramped to 220°C at a rate of 5°C min−1 for 1 min, then raised to 280°C at 15°C min−1, and held there for 10 min. The mass spectrometer was carried out in an electron impact (EI) source with 70 eV ionization energy in multiple reaction monitoring (MRM) mode at 1.3 kV. The temperatures for ion source, interface, and quadrupole were 230, 230, and 150 °C, respectively. The MS data were acquired in full scan mode from 40 to 500 amu. The temperatures for ion source, interface, and quadrupole were 230, 230, and 150°C, respectively. The retention time and MS of the compounds were compared with National Institute of Standards and Technology (NIST) mass spectrometry library to determine the types of fatty acids, and their contents were calculated by using an area normalization method.

#### Vitamin E Assay

The standard vitamin E was dissolved in n-hexane and formulated into a vitamin E standard solution with 5, 10, 25, 75, and 100 μg ml^−1^, respectively. After high-performance liquid chromatography Hitachi L-2000 (Shanghai, China) measurement, the standard curve was drawn by plotting the concentration of the vitamin E standard solution and the corresponding peak area. Then, 2 g of SWH seed oil was extracted by diethyl ether and evaporating the excess solvent, and the residue was dissolved in n-hexane and made up to metered volume. The test solution was filtered through a microporous membrane with 0.45 μm diameter. The content of vitamin E in seed oil was determined by the method of external standard peak area.

### Safety Evaluation of SWH Seed Oil

#### Cytotoxicity Assay

The MTT assay is a method commonly used to determine cytotoxicity, which is rapid, simple, and highly sensitive (25). Living cells can reduce MTT to produce blue crystals, which can be dissolved in DMSO and measured at 570 nm wavelength to obtain optical density (OD) value, which can reflect the number of living cells and determine the cytotoxicity.

The MTT solution (5 mg ml^−1^) was prepared by dissolving 0.5 g MTT in 100 ml phosphate buffer and filtering with aseptic membrane (0.22 μm diameter). Hela cells melt quickly in bain-marie at 37°C and centrifuged at 1,000 rpm for 5 min. Then Hela cells were cultured and represented in an RPMI 1640 medium containing 10% FBS. The 0.25% trypsin-treated cells with a concentration of 5 × 10^4^ cells were seeded into each 96-well plate with a volume of 0.2 ml for attachment to 80% over 24 h. In the next step, the old medium in each well was sucked out and then various samples (6.25, 12.5, 25, 50, 100, 200, 300, 400, and 500 μg ml^−1^; 200 μl.) dissolving in DMSO. The sample content in the wells was removed after 72 h of the incubation period. Then, 180 μl of RPMI 1640 medium with 10% FBS and 20 μl (5 mg ml^−1^ MTT) of MTT solution was added to each well and incubated for 4 h at 37°C. The supernatant was removed and 150 μl of DMSO was added to each well. The absorbance value was read at 570 nm. Medium without SWH oil was used as empty well control for absorbance reading. The percentage of surviving cells was calculated and determined using the following formula: surviving cells (%) = the absorbance value of the cells treated with SWH oil/the absorbance value of the untreated cells × 100% (mean ± SD, *n* =6).

#### Heavy Metal Content Assay

The content of heavy metals, such as copper, lead, chromium, zinc, cadmium, mercury, and arsenic in SWH seed oil was determined by atomic absorption spectrometry. The determination methods were based on GB/T 5009.13-2003, GB/T 5009.12-2010, GB/T 5009.123-2003, GB/T 5009.14-85, GB/T 5009.15-2003, GB/T 5009.17-1996, and GB/T 5009.11-1996, respectively.

### SWH Sample Screening Based on Genetic Diversity

#### DNA Extraction

Total genomic DNA was extracted from the seeds of SWH according to a modified hexadecyltrimethy ammonium bromide (CTAB) procedure. DNA concentration was estimated using electrophoresis on (0.8% (w v^−1^)) agarose gel, and diluted to 20 ng μl^−1^ with double distilled water for amplified fragment length polymorphism (AFLP) analysis.

#### AFLP Analysis

The genetic DNA was digested with MseI and EcoRI restriction enzymes combinations and ligated to MseI and EcoRI adapters simultaneously. The mixture of (DNA 4 μl (20 ng μl^−1^), EcoR I/MseI adapter 1 μl, EcoR I/MseI restriction enzymes 2 μl, 10×Reaction buffer 2.5 μl, ATP 2.5 μl, T4 DNA ligase 1 μl, and ddH_2_O 7 μl) was blended sufficiently and digested for 5 h at 37°C first, then for 4 h at 8°C, finally ligated overnight at 4°C. The restricted-ligated DNA was then diluted 20-fold with double distilled water to generate temperate DNA for amplification. Pre-selective amplification was performed in 25 μl reaction volume using E-A and M-C primer combinations. The reaction mixture consisted of 2 μl diluted restricted-ligated DNA, pre-amp mix 1 μl, dNTPs 1 μl, 10×PCR buffer 2.5 μl, Taq DNA polymerase 0.5 μl, and ddH_2_O 18 μl. Profile of PCR for pre-selective amplification was as followed: 28 cycles were performed at 94°C for 30 s, at 56°C for 30 s, at 72°C for 80 s, with a final extension at 72°C for 5 min. And products were diluted 20-fold with double distilled water as temperate DNA for selective amplification. Selective amplification by PCR was performed with eight EcoRI + 3 bases and MseI + 3 bases primer combinations. Profile of touchdown PCR for selective amplification was as followed: 13 cycles were performed at 94°C for 30 s, at 65°C (lowering the temperature by 0.7°C over the next cycles) for 30 s and at 72°C for 80 s, and then, 23 cycles were performed at 94°C for 30 s, at 55°C for 30 s, at 72°C for 80 s, with a final extension at 72°C for 5 min.

#### AFLP Data Analysis

AFLP products were analyzed automatically on an ABI PRISM 377 sequencer, with GeneScan-500 ROX as internal size standard, by 4% urea acrylamide gel electrophoresis at 1,200 V for 3 h. AFLP data were analyzed by GeneScan V3.1 software. AFLP profiles were converted into a presence/absence (1/0) data matrix using Binthere software. The 0–1 data matrix was further used to estimate genetic similarity between pairs of accessions according to DICE's similarity coefficient using NTSYSpc-2.11F software. In addition, the 0–1 data matrix was subjected to cluster analysis based on Euclidean distance to generate dendrogram with the help of PAST software. Besides, the 0–1 data matrix was performed to calculate the genetic relationship among all the accession.

### Breeding Experiments

#### Survey and Selection of Candidate Trees

Excellent germplasm should have robust and disease-free plants, upright and compact crown, numerous branches, short internodes, large and tidy fruiting volume. The seed yield per plant in bare land should be more than 2.5 kg, and in forest, 0.5 kg per plant was the lower limit. For the 4-year-old trees, their height, ground diameter, and the number of branches should be over 1.3 m, 4.5 cm, and 5, respectively. Through the analysis of morphological characteristics and AFLP results, the samples with strong growth potential, compact erect crown, early firmness, large and neat fruit ears, and strong resistance were screened.

#### Description of Experimental Site

The present experiment was conducted at Jiangshanjiao Experimental Forest Farm of Heilongjiang Academy of Forestry. The experimental site was on the slope with about 15 degrees in slope angle and face to southwest direction. The soil of experimental site was classified as sandy soil, and its soil thickness, topsoil thickness, total nitrogen, organic carbon, and hydrogen ion concentration were 45 cm, 25 cm, 0.96 g kg^−1^, 18 g kg^−1^, and 6.5, respectively.

#### Experimental Design and Measurements

These five samples were grown for 2 consecutive years with a randomized block design with 45 clusters per sample and three replicates. There were three plots in each row with protective plants at both ends of the line. The row spacing was 2 m × 3 m, and three rows of isolation protective plants were set on both sides of the garden. The hole size was 30 cm in depth and 40 cm in diameter. Besides, 5 kg of saprophytic farm fertilizer was applied to each hole and mixed with the topsoil to fill the bottom fertilizer. In the first year of planting, it was necessary to weed, expand the hole, and loosen soil to keep the soil moist. The survival rate of this year was 94.1%. In the second year, natural pollination was carried out. At the same time, the buds, thinning, and weak branches were pruned manually, and 3–8 main branches were left for each plant.

## Results

### Nutritional Value Assessment

As shown in Table 2, SWH seed oil contains 8 different fatty acids, among which polyunsaturated fatty acids (PUFA) and saturated fatty acids (SFA) account for 82.80 and 17.20% (PUFA:SFA = 4.8:1), respectively. The main components of PUFA in the oil are linoleic acid (50.56%), followed by linolenic acid (24.24%). Linoleic acid and linolenic acid are essential fatty acids which have health effects on the human body. The content of linoleic acid and linolenic acid in SWH seed oil is very high, accounting for 74.80% of the fatty acid, therefore, eating SWH seed oil is very beneficial to human health. The SFA includes palmitic acid (16.32%), 11-eicosanoic acid (3.71%), 9-octadecynoic acid (2.60%), 11,14-eicosodienoic acid (1.71%), arachic acid (0.51%), and tetradecanoic acid (0.36%).

**Table 2:** Fatty acids composition of SWH seed oil.

**Samples**	**Component**	**Molecular Formula**	**Relative Content (%)**	**Degree of Similarity (%)**	**Retention Time (min)**
1	Tetradecanoic acid (myristic acid)	C_14_H_28_O_2_	0.36	95	15.78
2	Hexadecanoic acid (palmitic acid)	C_16_H_32_O_2_	16.32	98	16.21
3	9,12-octadecadienoic acid (linoleic acid)	C_18_H_32_O_2_	50.56	99	16.62
4	9,12,15-octadecatrienoic acid (linolenic acid)	C_18_H_30_O_2_	24.24	99	17.19
5	9-octadecynoic acid (stearolic acid)	C_18_H_32_O_2_	2.60	99	19.23
6	11,14-eicosodienoic acid (ethyl linoleate)	C_20_H_36_O_2_	1.71	99	20.72
7	11-eicosanoic acid (gondoic acid)	C_20_H_38_O_2_	3.71	99	21.05
8	Arachic acid (eicosanoic acid)	C_20_H_40_O_2_	0.51	98	21.30

A standard curve was obtained by the peak area and concentration of VE. The linear regression equation for peak area (y) vs. concentration (x) is y = 2,440.4x−1,351.4, R = 0.9999. VE has a linear relationship in the range of 5–100 μg ml^−1^. Five parts of the same batch of SWH oil were used to calculate the VE content in the seed oil by external standard method. Based on the standard curve, the average content of VE in these five oil samples was calculated to be 25.92 mg kg^−1^.

### Safety Assessment

**Figure 2:**
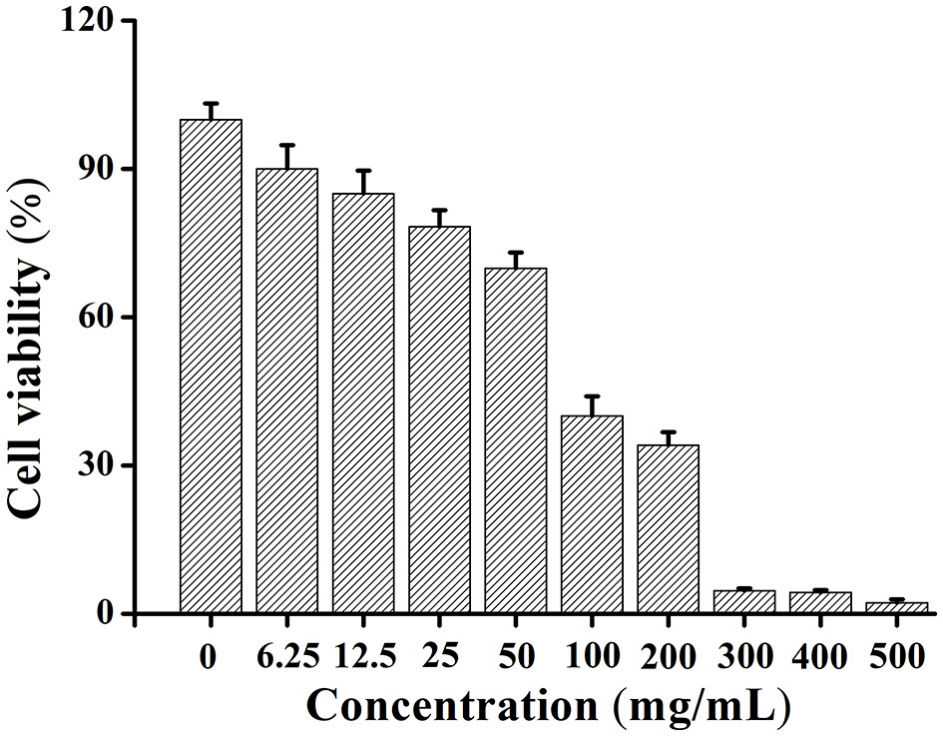
Cytotoxicity of SWH seed oil at different concentrations in Hela cells by MTT assay.

The cytotoxicity of SWH oil was determined by MTT assay, and the cell survival rate was calculated by evaluating the toxicity of SWH oil to Hela cells. Through comparing the survival rate of cells treated with SWH oil to the cells without being treated, the results showed that the cytotoxicity of SWH oil increased with the increase of concentration. The IC50 value of SWH oil for Hela cells was 88.82 μg ml^−1^ (Figure 2).

The heavy mental contents of SWH seed oil were analyzed by atomic absorption spectrometry. The contents were copper (0.035 mg kg^−1^), lead (0.0386 mg kg^−1^), zine (0.0769 mg kg^−1^), cadmium 0.00113 mg kg^−1^), and arsenic (<0.0005 mg kg^−1^). All of them were significantly lower than national standards (Table 3). Besides, there was no chromium and mercury have been detected.

**Table 3:** Heavy metal contents in SWH seed oil.

**Elements**	**Content (mg/kg)**	**National Standards (mg/kg)**
Copper	0.035	4
Lead	0.0386	1
Chromium	Not detected	—
Zinc	0.0769	5
Cadmium	0.00113	—
Mercury	Not detected	0.05
Arsenic	<0.0005	0.51

### Genetic Basis of SWH Breeding

#### Polymorphism Analysis and Similarity Index Analysis

**Figure 3:**
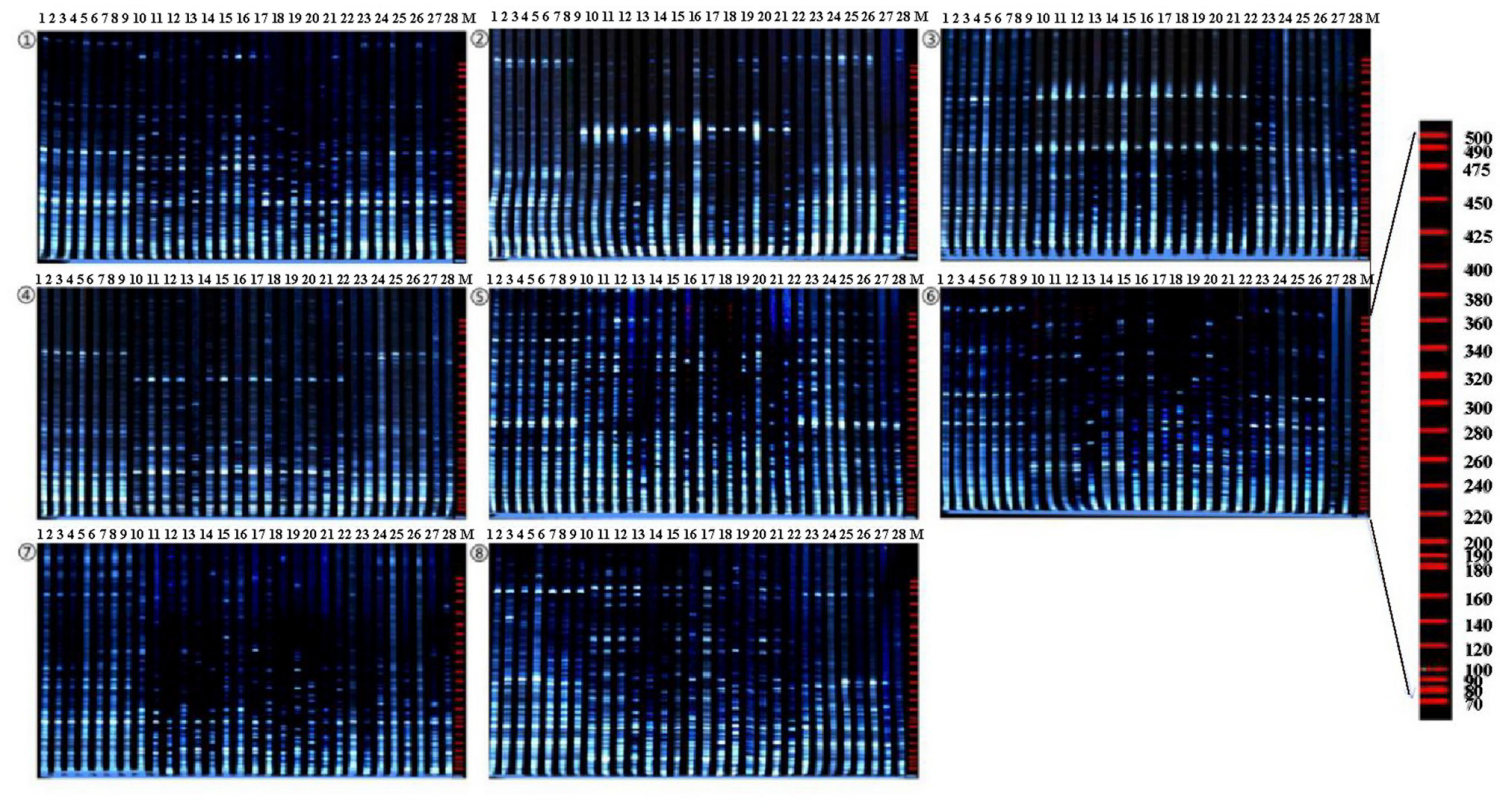
Amplified fragment length polymorphism (AFLP) profiles of 28 SWH accessions as revealed by 8 AFLP primer. Combinations. ①E-AAC/MCAC; ②E-AAC/M-CAG; ③E-AAC/M-CTC; ④E-AAG/M-CAC; ⑤E-AAG/M-CAG; ⑥E-ACA/M-CAG; ⑦E-ACA/M-CTC; ⑧E-ACG/M-CAC; Lane M stands for molecular weight marker.

Eight pairs of AFLP primer combinations which have high polymorphism and clear bands were selected for amplification and analysis of 28 accessions from 64 pairs of EcoRI/MseI primer combinations (Table 4 and Figure 3). A total of 705 fragments were scored across the 8 AFLP primer combinations assayed in this study, out of which 694 (98.4%) were polymorphic. The fragments generated ranged in size from 50 to 500 bp. The total number of fragments of each primer combination varied from 69 (E-ACA/M-CAG) to 104 (E-AAG/M-CAG) with an average of 88.1 fragments per primer combination. Polymorphic fragments ranged from 67 (E-ACA/M-CAG) to 104 (E-AAG/M-CAG) with an average of 86.75. The percent polymorphism varied from 95.2% (E-AAC/M-CAC) to 100% (E-AAG/M-CAC, E-AAG/M-CAG) with an average of 98.4% (Table 5). The experimental results showed that AFLP fingerprints of SWH have large variation and highly polymorphism. The overall mean similarity index for SWH accessions calculated based on all AFLP fragments amplified using Dice's similarity coefficient, ranged from 0.6649 to 0.8478. The highest similarity index (0.8478) was between the accessions of five and six, which were both collected from Heilongjiang Province. The lowest similarity index (0.6649) was found between the accessions of 3 and 14, which were collected from Heilongjiang and Hubei, respectively.

**Table 4:** Adapter sequence and primer combination.

	**Sequence (5^**′**^-3^**′**^)**
**Adaper**	
EcoRI	CTCGTAGACTGCGTACC
	AATTGGTACGCAGTCTAC
MseI	GACGATGAGTCCTGAG
	TACTCAGGACTCAT
**Primer**	
E-A/M-C	GACTGCGTACCAATTCA
	GATGAGTCCTGAGTAAC
E-AAC/M-CAC	GACTGCGTACCAATTCAAC
	GATGAGTCCTGAGTAACAC
E-AAC/M-CAG	GACTGCGTACCAATTCAAC
	GATGAGTCCTGAGTAACAG
E-AAC/M-CTC	GACTGCGTACCAATTCAAC
	GATGAGTCCTGAGTAACTC
E-AAG/M-CAC	GACTGCGTACCAATTCAAG
	GATGAGTCCTGAGTAACAC
E-AAG/M-CAG	GACTGCGTACCAATTCAAG
	GATGAGTCCTGAGTAACAG
E-ACA/M-CAG	GACTGCGTACCAATTCACA
	GATGAGTCCTGAGTAACAG
E-ACA/M-CTC	GACTGCGTACCAATTCACA
	GATGAGTCCTGAGTAACTC

**Table 5:** The number of polymorphic bands of amplified fragment length polymorphism (AFLP) fingerprints.

**Primer EcoR I/ Mse I**	**Number of amplified bands**	**Number of polymorphic bands**	**Polymorphism (%)**
AAC/CAC	83	79	95.2
AAC/CAG	103	102	99
AAC/CTC	90	89	98.9
AAG/CAC	98	98	100
AAG/CAG	104	104	100
ACA/CAG	69	67	97.1
ACA/CTC	76	75	98.7
ACG/CAC	82	80	97.6
Average	88.1	86.75	98.4
Sum	705	694	98.4

#### Principal Component Analysis (PCA) and Cluster Analysis

**Figure 4:**
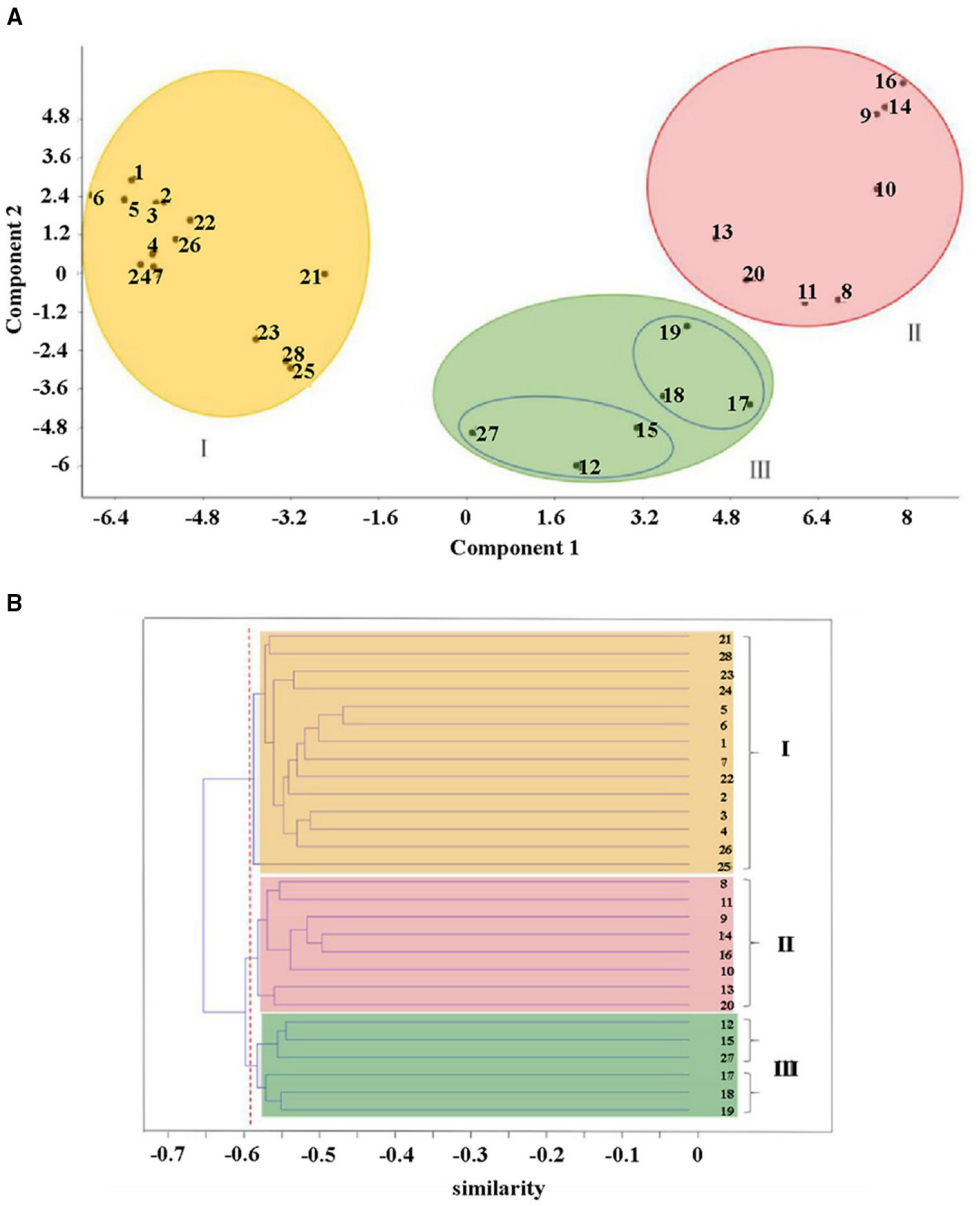
**(A)** Scatter diagram of principal component analysis (PCA) and **(B)** cluster analysis **(B)** of the AFLP results for 28 SWH accessions. I-III: the first to third genetic group, colored by yellow, red, and green, respectively.

Principal component analysis (PCA) showed a pronounced genetic variation among the 28 accessions of SWH. Results from the analysis revealed that the PC1 and PC2 encompassed 19.1 and 6.2% of the molecular variations, respectively. According to the two-dimensional plots, the 28 accessions generally dispersed into three-centered parts (Figure 4A). The dendrogram with −0.63 as the domain value showed three distinct groups (Figure 4B). These results were similar to the results of PCA analysis (Figure 4A). The first group contained 14 accessions, most of them collected from Heilongjiang province and the other two from Xinjiang and Jilin province. These three provinces are all located in northern China. The second group contained eight accessions collected from Shanxi, Anhui, Hubei, Henan, Shandong, Hebei, Liaoning, and Heilongjiang. They are mainly distributed in the central region and the coastal region in the east of China. The rest of six accessions of SWH in the third group collected from Jiangsu, Zhejiang, Fujian, Sichuan, Gansu, and Heilongjiang provinces.

### Breeding Analysis

#### Growth Characteristics of SWH

Based on the morphological characteristics and AFLP results, five samples were chosen (Table 6). In the first year, the plant grew slowly with only 1–3 sprouting branches and 20–50 cm in height. Some individuals began to form a small amount of flower buds and inflorescences. And the fruit setting rate is around 2.1%. In the second year, the growth of the stems accelerated, and the germination increased. The number of sprouting new branches reached 3–10 with more than 60 cm longest. And the fruit setting rate reached 32.1%. In the third year, the crown plexus was formed with 2.23 cm height, 4.63 cm diameter, and 0.94 m^2^ crown breadth. The fruit setting rate was more than 69.4%. The results showed that there were no significant differences in tree height and diameter between different samples. But the crown breadth, branch numbers, and the growth germination had a little bit of difference (Table 7). We can see that sample three had a strong growth potential, and its crown had a large number of branches. The average annual crown of the fourth year tree was 1.24 m^2^ and the plant height was 2.77 m. Besides, sample six had a large crown with many branches and a compact erect crown plexus. The average crown area of 4-years-old tree was 1.2 m^2^ and the plant height was 2.68 m.

**Table 6:** Main excellent characters of selected excellent samples.

**Samples**	**Tree age**	**Crown character**	**Fruit color**	**Ear character**
3	4	Compact	Bright red	Neat and beautiful
6	4	Moderate	Bright red	Neat and beautiful
14	4	Compact	Bright red	Neat and beautiful
20	4	Compact	Bright red	Neat and beautiful
27	4	Compact	Bright red	Neat and beautiful

**Table 7:** The phenotypic of selected samples.

**Samples**	**Plant Height (m)**	**Ground diameter (cm)**	**Crown breadth (m^**2**^)**	**Branch number (n)**	**Stem length (cm)**	**Stem diameter (cm)**	**Posture**
3	2.77	4.33	1.24	12	17.5	0.63	Compact, upright
6	2.68	3.60	1.2	10	15.1	0.65	Compact, upright
14	2.62	3.09	1.15	7	13.5	0.64	Compact, upright
20	2.44	3.2	1.23	5	14.2	0.64	Half open
27	2.35	3.1	1.21	6	13.0	0.61	Compact, upright

In terms of annual growth and development, the phenological period is generally consistent under the climatic conditions of Jiangshanjiao. In mid-March, the sap flowed and buds sprouted. The buds began to bloom from late March to mid-April. The florescence was in early May, and the young fruits appeared in mid-May. The fruit matured from mid-July to early August. In the middle and late August, the berries shrank and fell off, and the leaves fell in early October. The annual growth period was around 190 days (Table 8). Fruit development started from early May when the ovary was expanded to early August when the color of the fruits was changed completely and the fruits were matured (Table 8). The annual fruit development was ~90–100 days.

**Table 8:** Growth date of selected samples.

**NO**.	**Sprouting date**	**Squaring date**	**Leaf date**	**Florescence**			**Frutescence**	**Dead leaf date**	**Reproduction period per year / days**
				**Beginning date**	**Growing date**	**Ending date**			
3	19/3	24/3	15/4	29/4	1/5	8/5	17/7	1/10	192
6	20/3	26/3	16/4	29/4	3/5	10/5	20/7	5/10	195
14	21/3	25/3	18/4	28/4	5/5	9/5	22/7	3/10	192
20	20/3	24/3	16/4	29/4	1/5	11/5	25/7	8/10	197
27	22/3	26/3	18/4	28/4	4/5	12/5	27/7	6/10	194

#### Cultivation Yield Analysis

The average tree age of the samples was 4 years old and the average area of the experimental site was 42 m^2^. Besides, on average, the number of spikes in the sample was 1,204 and the seed weight was 4.868 kg and the crown projected area was 1.32 m^2^ (Table 9). Crown projection area is the main indicator for measuring the yield of different samples. The calculation shows that the actual average yield per hectare of the samples used in the experiment is 3,642 kg, which is significantly higher than the control group of 2,734.5 kg (Table 9).

**Table 9:** Yield factors and yield of superior tree comparative test lines.

**NO**.	**Tree age**	**Experimental site area / m^**2**^**	**Spike numbers/n**	**Seed weight / kg**	**Canopy projection area /m^**2**^**	**Average yield for three consecutive years**	
						**Crown projection yield / kg m^**−2**^**	**Area yield / kg**
3	4	48	1,354	6.16	1.24	0.62	275.7
6	4	36	1,082	4.32	1.2	0.60	266.8
14	4	48	1,336	4.88	1.15	0.53	235.7
20	4	48	1,161	4.64	1.23	0.47	209
27	4	42	1,085	4.34	1.21	0.51	226.8
Average	4	44.4	1203.6	4.868	1.206	0.546	242.8
Control group	4	48	1,211	4.32	1.32	0.41	182.3

The statistical analysis of canopy projection area was based on Excel and DPS software. It can be seen from the results that the yields of the sample three and six were significantly different from those of the other experimental samples (Table 10). According to ANOVA, the sum of square of deviations between and within groups were 0.2249 and 0.0047, respectively; the degree of freedom between and within groups was 6 and 14, respectively; the mean square between and within groups were 0.0408 and 0.0003, respectively.

**Table 10:** Crown projection yield of selected samples.

**Samples**	**Difference of crown projection yield / kg m^**−2**^**
3	0.625a ± 0.018
6	0.596a ± 0.008
14	0.536b ± 0.016
20	0.508b ± 0.017
27	0.4667c ± 0.003
Control group	0.415d ± 0.008

*Different letters indicate significant differences (P < 0.05)*.

## Discussion

### Nutrition and Safety of SWH

In terms of the GC-MS analysis of fatty acids showed that the content of unsaturated fatty acid in SWH seed oil was 82.8%. And linolenic acid accounts for 24.24%, while most of the edible oils contain almost no linolenic acid, such as peanut oil, olive oil, even soybean oil only with 8% linolenic acid (25, 26). Linolenic acid could help human beings enhance immunity, inhibit cancer, improve eyesight and memory, and enhance intelligence (27). The content of linoleic acid is 50.5%, which is higher than that of peanut oil with 26% and canola oil with 16.3%. Intaking moderate amounts of linoleic acid can effectively prevent cardiovascular and cerebrovascular diseases (28). Additionally, SWH oil contains vitamin E (25.92 mg kg^−1^), which can promote metabolism and anti-aging (29). Besides, based on the previous sensory evaluation of our group, SWH seed oil has excellent sensory qualities compared with existing edible oils in the market, especially when fried at high temperatures, which is superior to peanut oil and sesame oil. The flavor of SWH seed oil is as good as sesame oil and olive oil when it serves at medium and low temperatures. Therefore, SWH seed oil has edible value in terms of the detection of nutritional value and oil quality.

The IC50 value of SWH seed oil for Hela cells is 88.82 μg ml^−1^. If the IC50 value is <20 μg mL^−1^, indicating that the sample is with high cytotoxicity. The heavy metal content of SWH oil is all within the national standard range (Table 5). Therefore, SWH seed oil is considered to be a safe vegetable oil and has the potential to be developed into a healthy edible oil.

### Genetic Basis of SWH Breeding

In this study, the analysis of the genetic diversity of SWH offers a better understanding of the gene exchange and relationship in different regions of China. The eight AFLP primer combinations produced a total of 694 (98.4%) polymorphic bands were different in number, intensity, and position, indicative of a high level of genotypic variations among the investigated SWH accessions. At the same time, the low variation percentages (25.3%) of the first two components in the PCA analysis could also be attributed to high genetic variability of SWH (30). This may be explained that SWH could adapt to complex living environments, such as different climates, humidity, and soil types, easily (31). In addition, the high variability of SWH is crucial for utilizing and improving unique characteristics of the germplasm resources, and for facilitating future selection and recombination cultivating strategies (32).

According to cluster analysis and PCA analysis, the 28 materials are mainly divided into three large groups. The first category is mainly the provenance from Heilongjiang province in northeastern China. The fruit color of the first category is all bright red and the crown forms are compact or moderate. This group has high similarity indices from 0.7315 to 0.8478, meaning that their genetic distance is not far. We inferred that SWH from the same province are genetically closely related and they have potential to be substitutes for each other in future crossbreeding. In the meantime, researchers should pay attention to avoid inbreeding depression if the parents of hybridization have little difference in geographical variations (33). The second major group is scattered in various provinces in China, mainly in the central and eastern coastal regions, and includes all crown types, such as compact, moderate, and open.

The fruit color of the group is not only bright red but also purple red, indicating that this group has higher variability than the first group. Meanwhile, this group included one accession (26) collected from Heilongjiang province. This may be explained by that the SWH in most parts of China is introduced and cultivated by this species. In addition, perhaps, this species has strong resistance and can adapt to different climate conditions. Thus, sample 20 might be one of the candidate worthies of widespread cultivation. The third group includes accessions from both southeastern and western regions of China, and also contains one accession (33) gathered from Heilongjiang province. Therefore, it is considered that the remaining of SWH in this group evolved from the species, which is highly adaptable like accession (26) in the second group. It is also reported that high degree of divergent genotypes is expected to produce more promising and desirable re-combinations in the subsequent generations for achieving maximum genetic advance (34). The fruit color of samples 12 and 15 in the third group is special purple black which is different from the purple red in the remaining samples. These two samples might be used to enrich SWH fruit morphology in hybridization breeding. The third group has different types of mixed crowns. According to the similarity indices, accessions 3 and 14, which have been screened in cultivation experiment due to excellent external characters, were the most divergent (0.6649) and accordingly might have been a great eventuality of heterosis in a breeding direction aimed to improve the desired traits (35).

### Plant Breeding

The cultivation experiment found that the common characteristics of the five excellent strains include large amounts of fruit yield, bright red fruit, large and neat fruit ears, and high seeding rate. The average yield per hectare of these five samples was 3,642 kg, and the seed yield was 33.2% higher than that of the control group. It is estimated that the canopy projection area of mature SWH tree could reach about 4.5 m^2^. In addition, we can see that sample 3 and 6 have high seed yield, with 4,135.5 and 4,002 kg ha^−1^, respectively. And these data are higher than normal yield (2,902.5 kg) of 42.59 and 33.33%, respectively. The average plant height of the 4-year-old plants was 2.77 and 2.68 m, respectively for sample three and six. Besides, it can be seen that the crown projection yield of sample three and six significantly exceeded sample 14, 20, 27, and control group, which indicates that these two strains are highly productive (36). We considered that these two samples are suitable for dense planting. We predicted that if samples 3 and 6 were used to establish SWH seed oil raw material forest, the average grain yield per hectare could reach ~4,500–5,350 kg.

*Sambucus williamsii* Hance seed oil is a high-quality edible oil with rich linolenic acid. Its seed residue is rich in essential amino acids, protein, calcium, and other nutrients, which are extremely suitable for patients with diabetes, cardiovascular, and cerebrovascular diseases and early fracture healing to eat. The fruit can be made into fruit juice, fruit wine, and jam. At the same time, the leaves of SWH can be developed as sprouts or bud tea because it has the characteristics of hypertrophy, wide length, fresh leaf buds, good flavor, and high nutrient content. Therefore, we predict that the SWH by-products have strong economic, social, and ecological benefits.

The data showed that there was no significant difference in the tree height among different samples. However, there were certain differences in the size of the crown, the number of branches, and the growth of new branches, which meant that there were many excellent strains of SWH for screening. They have wide cultivation adaptability with stable fruit quality and genetic traits (37). If appropriate light, water, and nutrient conditions are given, the cultivation of SWH seed oil raw material forest has great development potential. However, the above-mentioned excellent samples have not yet entered the delivery period. The soil fertility of the test ground is poor, and the field management level is general, which indicates that it has the potential for increasing production. In addition, some excellent clones still need to be further improved due to short breeding time, few materials, and data.

## Data Availability Statement

The original contributions presented in the study are included in the article/supplementary material, further inquiries can be directed to the corresponding authors.

## Author Contributions

SW: investigation and writing. YY and MC: methodology and data curation. KehL and KewL: supervision, project administration, and funding acquisition. All authors have read and agree to the published version of the manuscript.

## Funding

This research was funded by the National Natural Science Foundation, China (81572989) and the Research Project of Heilongjiang Forest Industry (sgzjY2014021).

## Conflict of Interest

The authors declare that the research was conducted in the absence of any commercial or financial relationships that could be construed as a potential conflict of interest.

## Publisher's Note

All claims expressed in this article are solely those of the authors and do not necessarily represent those of their affiliated organizations, or those of the publisher, the editors and the reviewers. Any product that may be evaluated in this article, or claim that may be made by its manufacturer, is not guaranteed or endorsed by the publisher.
